# Long-Term Assessment of the In Vitro Corrosion Resistance of Biomimetic ACP Coatings Electrodeposited from an Acetate Bath

**DOI:** 10.3390/jfb12010012

**Published:** 2021-02-07

**Authors:** Patrycja Osak, Joanna Maszybrocka, Julian Kubisztal, Patryk Ratajczak, Bożena Łosiewicz

**Affiliations:** Faculty of Science and Technology, Institute of Materials Engineering, University of Silesia in Katowice, 75 Pułku Piechoty 1A, 41-500 Chorzów, Poland; joanna.maszybrocka@us.edu.pl (J.M.); julian.kubisztal@us.edu.pl (J.K.); patrykrataj@interia.pl (P.R.)

**Keywords:** amorphous calcium phosphate, artificial saliva, corrosion resistance, titanium

## Abstract

Calcium phosphate coatings are able to improve the osseointegration process due to their chemical composition, which is similar to that of bone tissues. In this work, to increase the long-term corrosion resistance and to improve the osseointegration process of commercially pure titanium Grade 4 (CpTi G4), biomimetic amorphous calcium phosphate (ACP) coatings were electrodeposited for the first time from an acetate bath with a pH level of 7.0 and a Ca:P ratio of 1.67. ACP coatings were obtained on CpTi G4 substrate subjected to sandblasting and autoclaving using electrochemically assisted deposition at a potential of −3 V relative to the open circuit potential for 30 min at room temperature. SEM, EDS, 2D roughness profiles, amplitude-sensitive eddy current method, and Kelvin scanning probe were used for the surface characterization of the biomaterial under study. In vitro corrosion resistance tests were conducted for 21 days in artificial saliva using open circuit potential, polarization curves, and electrochemical impedance spectroscopy measurements. The passive-transpassive behavior was revealed for the obtained ACP coatings. The long-term corrosion resistance test showed a deterioration of the protective properties for CpTi G4 uncoated and coated with ACP with immersion time. The mechanism and kinetics of the pitting corrosion on the CpTi G4|TiO_2_|ACP coating system are discussed in detail.

## 1. Introduction

Titanium is one of the vital elements. This metal shows high biocompatibility with living tissue and high corrosion resistance, which is related to the presence of a self-passive oxide layer (TiO_2_) with a thickness of about 2–10 nm on its surface [1]. These unique properties of titanium make it widely used in modern implantology, especially for the production of dental implants. In medicine, titanium containing alloying additives such as Al, V, Nb, Ta, Zr, Mo, and Hf is used [2]. Depending on the degree of purity, i.e., the content of other elements, titanium can be divided into different purity classes. Grade 1, 2, 3, and 4 titanium belongs to the commercially pure (cp) group as the titanium content is over 99%. From Grade 5 of purity, Ti belongs to titanium alloys due to the content of alloying elements, usually in the form of aluminum and vanadium. For the production of dental implants, titanium of Grade 4 is most often used due to the mechanical strength of about 550 MPa and Young’s modulus of about 104 GPa [3,4].

The increase in the standard of living of the society and the growing need to protect health, particularly the field of implantology, require the use of new generations of biomaterials. The dynamic development in the field of implant prosthetics is mainly due to the intensive development of material engineering. The growing population of toothless people makes it necessary to use dental implants to restore the functions of the masticatory system, which in turn prevents further changes in the stomatognathic system. Nowadays, implantoprosthetic treatment is considered the most physiological treatment. Titanium dental implants are made in the form of a screw with a cylindrical or conical shape. Current trends in the development of implantology aim at the elimination of toxic alloy additives such as Ni, V, and Co. The ions of these metals, being a product of corrosion, can cause allergies and disrupt brain’s ability to function, which in turn leads to numerous diseases. Replacing tissues with biomaterials requires them to meet stringent criteria regarding chemical composition, corrosion resistance, and mechanical strength. In addition, many efforts are being made to improve the physicochemical properties of the materials used, which will allow for the longest possible trouble-free functioning in the oral cavity [1,2].

Over the years, the surface of titanium dental implants has been modified. The composition and type of the implant surface layer are of greatest importance for the success of the osseointegration process. In the 1970s, titanium dental implants with a smooth surface were developed. However, smooth machine implants have the lowest degree of osseointegration due to the low surface development [5]. For titanium implants, the most commonly used surface is the sandblasted surface [6,7]. Currently, implant surfaces coated with titanium plasma (TP) and hydroxyapatite (HA) are also used [7,8]. Double acid-etched (DE) surfaces and a combination of sandblasting and surface etching (sandblasted large-grit acid-etched, SLA), are also used. The SLA surface can be kept in NaCl solutions to obtain a hydrophilic surface called SLActive [9]. The TiUnite surface of dental implants, which is obtained by anodizing, is also known [10].

The surfaces of dental implants can be modified by applying ceramic and/or polymer coatings, as well as oxide layers. The purpose of modifying the surface of dental implants is to accelerate the process of osseointegration and to reduce complications related to implant-prosthetic treatment. For this purpose, implant surfaces are modified using biomimetic coatings with a composition similar to that of bone tissues. It is best to use calcium phosphate (CaP) coatings, which improve the process of osseointegration [11]. Currently, CaP bioceramics are widely used in medicine, particularly in orthopedics, plastic surgery, and dentistry. The CaP coatings on the surface of the implants support osteoinduction by increasing the levels of calcium and phosphorus ions in the surrounding tissues, which leads to the induction of natural apatite (Ap) on the surface of the implant. Amorphous calcium phosphate (ACP) is a precursor of HA and is involved in the transition phase of mineralization, particularly of the dentin matrix protein (DMP1) [12]. ACP is a unique form of CaP minerals found in organisms [13,14,15]. It is widely used in dentistry due to its excellent bioactivity and adjustable biodegradation rate. ACP has also been shown to increase mesoblast alkaline phosphatase activity, improve cell proliferation, and promote cell adhesion [13]. Clinical trials of ACP indicate the good properties of this material for conversion to bone apatite in vivo, making it a seemingly excellent new class material for bone defect replacement and repair [12]. Today, ACP is used as a precursor to more advanced bone structures [16].

The oral cavity’s biological environment varies due to its anatomical structure, physiology, and biochemical processes. Biochemical functions occur in the oral cavity, including the secretion of saliva and gingival crevicular fluid [17,18]. The oral cavity has a permanent microflora that is entirely dependent on the individual. When the environment is disturbed, microorganisms appear. Inflammation, prosthetic restorations, and tooth extractions influence the oral cavity. Teeth accumulate large amounts of bacteria in the form of dental plaque, also known as bacterial plaque. Dental plaque naturally builds up in the oral cavity as a result of tooth decay and other periodontal diseases. The dental plaque mineralizes to form tartar, consisting of 20% of the elements that make up plaque and 70% of calcium phosphate and calcium carbonate. A layer of live bacteria covers the tartar [6]. As dental plaque increases, the risk of gingivitis increases. Bacterial metabolic products that settle on tartar affect bone tissue’s resorption, which is associated with tooth loss [19,20]. Saliva consists mainly of water (94–99%), proteins, glycoproteins, and lipids. Saliva also consists of sodium, potassium, chloride, calcium, phosphate, and carbonate ions, the most aggressive of which are chloride ions which cause pitting corrosion of dental implants [20].

One of the basic criteria for assessing the quality of new ACP coatings is their long-term behavior in the biological environment. In vitro tests of corrosion resistance of ACP coatings are necessary before proceeding with further tests on living organisms [21,22]. Corrosion processes on the titanium surface in saliva are of an electrochemical nature. For this reason, electrochemical methods are an appropriate research tool for the characteristics of the corrosive behavior of biomaterials. Among the numerous electrochemical methods, electrochemical impedance spectroscopy (EIS) is considered special, as it is the only AC method that allows the determination of the mechanism and kinetics of electrochemical corrosion with simultaneous characteristics of the capacitive behavior of titanium, the protective layer, and the saliva environment system [23,24,25,26].

This work mainly focuses on the long-term EIS diagnosis on the corrosion resistance of the sandblasted and autoclaved CpTi G4 covered with biomimetic ACP coatings obtained by electrochemically assisted deposition (ECAD) from a newly developed acetate bath with a Ca:P ratio of 1.67. The deposition bath with pH 7 was composed of calcium acetate, ammonium hydrogen phosphate, isocitric acid, and ammonium chloride. The advantage of the ECAD method used was its high repeatability and the possibility of deposition at room temperature and neutral pH, which allowed us to co-deposit inorganic and organic components. The proposed acetic bath can be an alternative to acidic ACP deposition baths. The electrode and electrolyte interfacial properties in artificial saliva solution (ASS) were determined using the equivalent electrical circuit concept.

## 2. Materials and Methods

### 2.1. Substrate Preparation

A CpTi G4 rod with a diameter of 10 mm (Bibus Metals, Dąbrowa, Poland) was cut into disk-shaped samples which were 5 mm high. CpTi G4 disks were mechanically pre-polished on 600 to 5000 # grit SiC papers. A colloidal silica suspension with a grain size of 0.04 µm (OP-S suspension, Struers, Cleveland, OH, USA) was used in the final step of the mechanical polishing. Samples with a mirror surface were cleaned in an ultrasonic cleaner for 20 min in acetone, and then in ultrapure water with a resistivity of 18.2 MΩ cm at 25 °C, produced with a Milli-Q^®^ Advantage A10 Water Purification System (Millipore SAS, Molsheim, France). The polished CpTi G4 samples were sandblasted with a white Al_2_O_3_ FEPA Grit F220 abrasive [27]. Next, they were sonicated again. After drying, the samples were autoclaved using the Zealway Model GR60 DA (Xiamen, China). A detailed description of the conditions of the sandblasting and autoclaving process is given in the earlier work [28].

### 2.2. ECAD of CaP Coatings

The CaP coatings were deposited on a sandblasted and autoclaved CpTi G4 substrate meeting the requirements of ASTM F67-13 [29] and ISO 5832-2 [30]. The ECAD process was used to obtain the CaP coatings in the acetate bath consisting of C_4_H_6_CaO_4_—2.94 g dm^−3^, (NH_4_)_2_HPO_4_—1.32 g dm^−3^, C_6_H_8_O_7_—2.00 g dm^−3^, and NH_4_Cl—2.00 g dm^−3^. The content of the bath components provided a Ca:P molar ratio of 1.67. A 30% NH_3_ solution was used to adjust the pH of the acetate bath to 7.0(1). Reagents of recognized analytical grade (Avantor Performance Materials Poland S.A., Gliwice, Poland) and ultrapure water were used to prepare the bath.

A single-chamber electrochemical cell with a 3-electrode system was used for the deposition of CaP coatings, in which CpTi G4 of 0.785 cm^2^ was the cathode, the platinum foil of 10 cm^2^ was the anode, and the reference electrode was a saturated calomel electrode (SCE) inserted into the bath using a Luggin capillary filled with the same solution. The method of cathode preparation was described in detail by the authors of [25]. Immediately before deposition, the CpTi G4 substrate was chemically activated in a 4% NaOH solution for 5 min. In the first stage of the ECAD, the open circuit potential (*E*_OC_) was measured for 30 min in the acetate bath at room temperature using the electrochemical system of Autolab/PGSTAT30 (Metrohm Autolab B.V., Utrecht, The Netherlands). In the second ECAD stage, chronoamperometric measurements were carried out at a potential of −3 V relative to the *E*_OC_ for 30 min. The ECAD-derived CaP coatings were rinsed using ultrapure water and dried in air for 24 h.

### 2.3. Materials Characterization

The surface morphology, chemical composition, and surface distribution of Ca, P, and Ti were studied using the JEOL JSM-6480 scanning electron microscope (SEM, Peabody, MA, USA), equipped with an energy dispersion spectroscopy (EDS) attachment. For each tested sample, 10 SEM images were taken.

The thickness of the CaP coatings was tested by the amplitude-sensitive eddy current method using the Dualscope FMP20 gauge (Helmut Fischer GmbH, Sindelfingen, Germany) with a probe FTA 3.3 (Helmut Fischer GmbH, Sindelfingen, Germany). Calibration was performed using the CpTi G4 substrate and 2 standard foils with a thickness of 24.3(5) and 48.2(1) μm (Helmut Fischer GmbH, Sindelfingen, Germany). For each sample, 30 thickness measurements were performed.

The surface roughness tests were carried out using a Mitutoyo Surftest SJ-500/P profilometer (Mitutoyo Polska Ltd., Wrocław, Poland). Measurements of changes in the surface profile were recorded 5 times for each sample on a sampling length of approximately 10 mm with a step of 0.1 μm and a speed of 200 μm s^−1^. In accordance with ISO 4287 [31], the recorded surface texture parameters were processed and developed using the FORMTRACEPAK computer program.

In each series of measurements, 10 samples were tested. The obtained results are presented as the mean value with standard deviation (*SD*).

### 2.4. In Vitro Corrosion Resistance Measurements

The long-term in vitro tests of corrosion resistance were conducted in de-aerated artificial saliva solution (ASS) of pH = 7.4(1) at 37(1) °C. The ASS was composed of NaCl—0.70 g dm^−3^, KCl—1.20 g dm^−3^, Na_2_HPO_4_—0.26 g dm^−3^, NaHCO_3_—1.50 g dm^−3^, and KSCN—0.33 g dm^−3^. A solution of 4% NaOH and 1% C_3_H_6_O_3_ was used to adjust the pH according to ISO 10,271 [32]. The configuration of the electrochemical cell was described in detail in our earlier works [23,28]. All potentials were recorded against the SCE.

The *E*_OC_ measurements were carried out for 21 days of immersion in ASS. The EIS spectra were recorded at the *E*_OC_ every 24 h in the range of frequency (*f*) from 10^4^ to 10^−3^ Hz using a sine wave of 10 mV amplitude. The analysis of the recorded EIS data was performed based on the equivalent electrical circuit concept using the complex nonlinear least squares (CNLS) method. In the CNLS fitting procedure, the EQUIVCRT program based on the Boukamp code was used (Metrohm Autolab B.V., Utrecht, The Netherlands) [33]. 

The anodic polarization curves were obtained on days 1 and 21 of the test, starting from a potential of 150 mV more negative with respect to the *E*_OC_ to 4 V with the *v* = 1 mV s^−1^.

For each type of electrode, three measurement series were performed, and the determined parameter values were given as average values with *SD*.

### 2.5. Work Function Measurements

Work function (*WF*) maps of the sandblasted and autoclaved CpTi G4 with and without CaP coatings were recorded before and after 21 days of immersion in artificial saliva using Scanning Kelvin Probe (SKP) method and PAR Model 370 device (Princeton Applied Research, Oak Ridge, TN, USA). The scanning area was 1 mm^2^ and the distance between the sample and the tungsten probe (ø150 μm) was ca. 90 µm. The work function of the sample (*WF*_sample_) was determined using the equation *U*_cpd_ = (*WF*_sample_ − *WF*_probe_)/e where *U*_cpd_ is the measured contact potential difference, *WF*_probe_ is the work function of the probe, and e is the elementary charge. Statistical analysis of the *WF* maps allowed us to determine parameters that quantitatively described the electric properties of the material surface, i.e., the arithmetic average (*WF*_av_), arithmetic mean deviation (*WF*_a_), root mean square deviation (*WF*_rms_), skewness (*WF*_sk_), and kurtosis (*WF*_ku_). The detailed procedure for the determination of the parameters can be found elsewhere [34].

## 3. Results and Discussion

### 3.1. Microstructure Study

**Figure 1:**
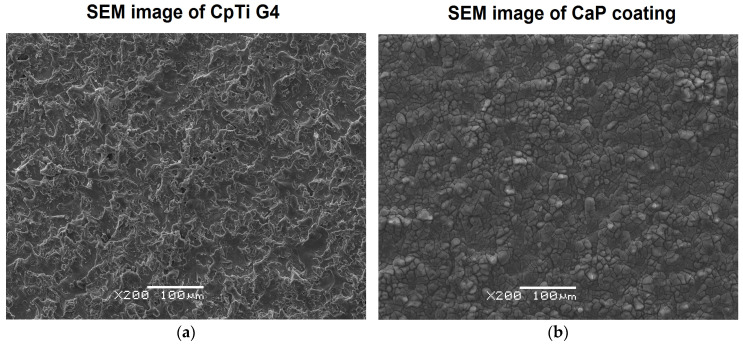

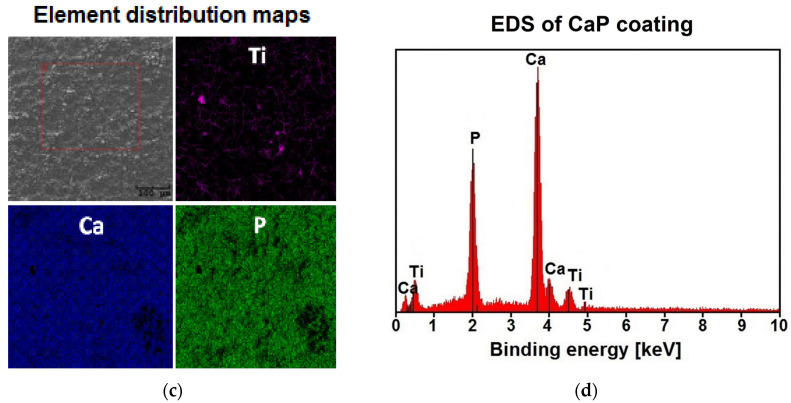
(**a**) SEM image of the surface morphology of commercially pure titanium Grade 4 (CpTi G4) substrate; (**b**) SEM image of the calcium phosphate (CaP) coating deposited at −3 V for 30 min at 20 °C; (**c**) SEM image of a selected microregion of CaP coating with the corresponding EDS map of Ti, Ca, and P element distribution; (**d**) Energy dispersive spectrum in the microregion of CaP coating.

The ECAD process, conducted at the potential value of −3 V at the porous titanium substrate (Figure 1a), produced a continuous CaP coating with a thickness of 11.3(7) µm, which fully covered the titanium surface and adheres well to the substrate (Figure 1b). The surface morphology in the selected microregion of the ECAD-derived CaP coating with corresponding maps of the distribution of chemical elements is shown in Figure 1c. Each map is marked with a different color to locate elements originating from the coating as Ca (blue) and P (green) and from the Ti substrate (purple). These maps indicate that, in the observed microregion, the Ca and P elements were evenly distributed on the surface of the coating, with Ca being more abundant. A subtle response from the substrate indicating microcracks in the CaP coating was also observed. The EDS spectra were collected from five microregions. Figure 1d shows a representative energy dispersive spectrum in the microregion of the coating. Based on the binding energy of the characteristic peaks, the presence of Ti coming from the substrate and the elements forming the CaP coating were identified. The element concentration from the peaks were determined, and the ratio of Ca:P = 1.5 was revealed (Table 1). The obtained result indicates that the deposited calcium phosphate was ACP. This is consistent with our X-ray structural analysis, which revealed the amorphous nature of the CaP coating deposited on the titanium surface under the same conditions [35]. Such a biomimetic coating may play an important role in bone biomineralization processes.

### 3.2. Surface Roughness Study

**Figure 2:**
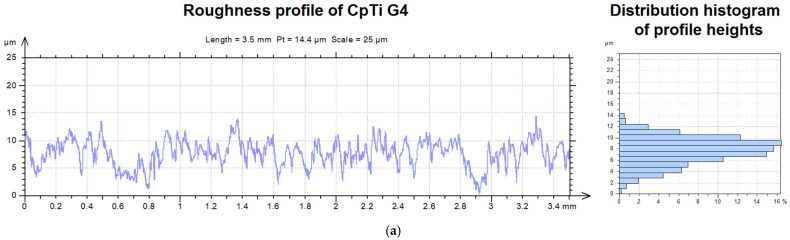

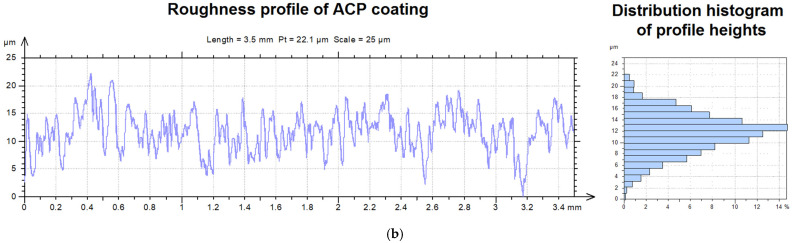
The roughness profile and the distribution histogram of the profile heights: (**a**) CpTi G4 substrate subjected to sandblasting and autoclaving; (**b**) ACP coating deposited at −3 V for 30 min at 20 °C.

An example of the roughness profile for CpTi G4 substrate subjected to sandblasting and autoclaving and ECAD-derived ACP coating is shown in Figure 2.

The osseointegration process depends on many factors, including the quality of the bone, biocompatibility, and surface roughness of dental implants with *R*a between 1 and 3 [36]. The arithmetic average height parameter, defined as the average absolute deviation of the roughness irregularities from the mean line over one sampling length, was almost 1.5-times larger for the ACP coating (Figure 2b) than that determined for the titanium substrate (Figure 2a), and equaled 1.91(11) μm and 2.81(8) μm, respectively. In the case of root mean square roughness parameters, which corresponds to the standard deviation of the height distribution, defined on the sampling length, the value increased after ACP coating deposition from 2.38(12) μm to 3.56(22) μm. The value of skewness parameters indicates that the bulk of the material of the sample was above (negative skewed) and below (positive skewed) the mean line for the CpTi G4 sample and ACP coating, respectively, as shown in Figure 2. There was also an increase in the relative length of the profile parameter from 5% to 6% for the titanium substrate and ACP coating, respectively. 

### 3.3. Electrochemical Impedance Spectroscopy Study

**Figure 3:**
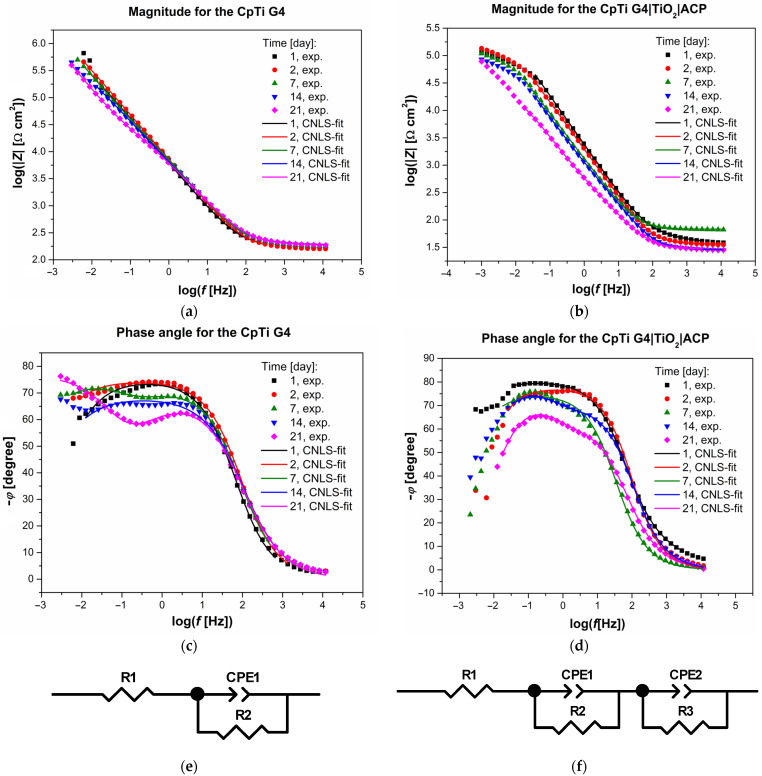
Bode diagrams for the sandblasted and autoclaved CpTi G4 and CpTi G4|TiO_2_|ACP electrodes in the artificial saliva solution (ASS) solution at 37 °C with the equivalent electrical circuit models for the pitting corrosion process used for CNLS-fitting: (**a**) Magnitude for the CpTi G4; (**b**) Magnitude for the CpTi G4|TiO_2_|ACP; (**c**) Phase angle for the CpTi G4; (**d**) Phase angle for the CpTi G4|TiO_2_|ACP; (**e**) One-CPE model; (**f**) Two-CPE model. Symbols are experimental data and continuous lines are CNLS fit.

The experimental Bode diagrams for the CpTi G4 and CpTi G4|TiO_2_|ACP electrodes recorded in artificial saliva solution at 37 °C for 21 days are displayed as symbols in Figure 3a–d. The dependence of log|*Z*| = log(*f*) in the midfrequency range showed a slope of about −1 (Figure 3a,b). The higher values of log|*Z*| in the entire range of tested frequencies were observed for the CpTi G4 substrate as compared to the CpTi G4|TiO_2_|ACP electrode. In the case of both tested materials, the highest values of log|*Z*| corresponding to the highest corrosion resistance were recorded after the first day of immersion. The onset of the pitting can be found from a decrease in log|*Z*|, which was especially noticeable at low frequencies below 0.1 Hz.

The dependence of the phase angle (*φ*) as a function of log *f* is shown in Figure 3c,d for the CpTi G4 and CpTi G4|TiO_2_|ACP, respectively. The Bode diagrams of the phase angle recorded after the first and second day of immersion showed a plateau in the mid-frequency range, which confirmed the strong barrier properties of the electrode surface. The maximum values of *φ* were below −80°. For the CpTi G4 electrode, only one time constant in the electrical circuit was observed for 21 days of immersion (Figure 3c), which indicates a stable electrode surface. In the case of the CpTi G4|TiO_2_|ACP electrode, one time constant in the electrical circuit was present only for the first 7 days. Then, from day 8 to day 21 of immersion, two time constants were observed in the electrical circuit. At the same time, the value of *φ* in the entire range of tested frequencies underwent significant changes with the immersion time. This indicates that the electrode surface was not stable during the corrosion test. Such long-term impedance behavior characterizes titanium and its alloys coated with passive layers in a biological milieu [25,37,38]. The experimental high values of |*Z*|*_f_*_→0_ and *φ*, presented in Figure 3a–d, are typical for biomaterials with capacitive behavior and high corrosion resistance [1,23,24,25,28,37,38,39,40].

**Figure 4:**
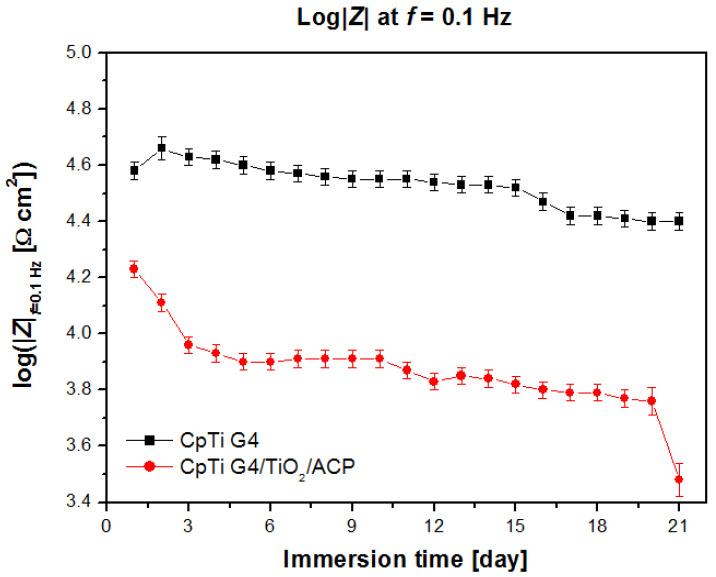
The dependence of the impedance module log|*Z*| at *f* = 0.1 Hz from immersion time for the sandblasted and autoclaved CpTi G4 and CpTi G4|TiO_2_|ACP electrodes in the ASS at 37 °C.

The parameter log|*Z*|*_f_*_=0.1Hz_ can be used for the comparative assessment of in vitro corrosion resistance of the tested materials (Figure 4). Higher values of log |*Z*|*_f_*_=0.1Hz_ were observed for the CpTi G4 electrode covered with a passive oxide layer. A decrease in log |*Z*|*_f_*_=0.1Hz_ with immersion time is indicative of deterioration of the protective properties for both the CpTi G4 and CpTi|TiO_2_|ACP electrodes. However, both tested materials still exhibited anticorrosion properties. These results indicate that the ACP coatings formed on the surface of the CpTi G4 electrode and, as a result of the ECAD process, became thicker and more porous with immersion time. It can be assumed that the coatings consisted of a porous outer layer and a dense inner layer, which acted as a barrier layer against corrosion. This long-term behavior is similar to the previously noted behavior of passivated Ti alloys in solutions containing chlorides which initiated pitting corrosion [25,37,38].

The EIS experimental data on the protective properties of the surface of the sandblasted and autoclaved CpTi G4 and CpTi G4|TiO_2_|ACP electrodes, recorded during the first 7 days of immersion, were approximated using the equivalent electrical circuit model for the pitting corrosion process which is called one-CPE model (Figure 3e). This model with four adjustable parameters, *R*_1_, CPE-*T*_1_, CPE-*ϕ*_1_, and *R*_2_, displays only one semicircle on the Nyquist plot [41]. The construction of this equivalent electrical circuit and the physical meaning of the individual circuit parameters have been discussed in detail in our previous works [23,24,28,39,41]. 

To approximate the experimental EIS data recorded for the CpTi G4|TiO_2_|ACP electrode from day 8 to day 21 of immersion, the equivalent electrical circuit model for the pitting corrosion process shown in Figure 3f was used. This two-CPE model is described by seven adjustable parameters as *R*_1_, CPE-*T*_1_, CPE-*ϕ*_1_, *R*_2_, CPE-*T*_2_, *ϕ*_2_, and *R*_3_, and displays two semicircles on the Nyquist plot [39,41]. In this model, the presence of a two-layered structure of the passive film on the surface of the metallic electrode was assumed. The semicircle at high frequencies (HF) refers to the outer layer with a porous structure and is described by the circuit parameters *R*_1_, CPE-*T*_1_, CPE-*ϕ*_1_, and *R*_2_. The remaining parameters of the circuit, namely CPE-*T*_2_, CPE-*ϕ*_2_, and *R*_3_, describe the second semicircle at low frequencies (LF), which refers to the inner layer directly adjacent to the substrate and showing strong barrier properties.

Figure 3a–d illustrates the CNLS-fitted data marked as continuous lines which were obtained using the electrical equivalent circuit shown in Figure 3e,f, respectively. The very good quality of the CNLS-fit is visible. All CNLS-fit parameters determined using the equivalent electrical circuit model displayed in Figure 3e are summarized in Table 2 for the sandblasted autoclaved CpTi G4 electrode. Table 3 presents all CNLS-fit parameters determined for the CpTi G4|TiO_2_|ACP electrode using the equivalent electrical circuit models for pitting corrosion process shown in Figure 3e,f.

The values of the *R*_2_ parameter had higher values in the case of the CpTi G4 electrode, which indicates a greater resistance to the charge transfer in the ongoing process of electrochemical corrosion (Table 2). The value of *R*_2_ determined for the sandblasted and autoclaved CpTi G4 electrode decreased from 2.52(47) × 10^6^ Ω cm^2^ after the first day of immersion to 9.30(90) × 10^4^ Ω cm^2^ on the last day of the corrosion test. For the CpTi G4|TiO_2_|ACP electrode, the charge transfer resistance associated with the outer oxide layer, *R*_3_, was as much as 677- and 170-times smaller than *R*_2_ after days 14 and 21 of immersion, respectively. This indicates that there were much stronger barrier properties of the inner oxide layer directly adjacent to the titanium substrate which provided essential protection against corrosion (Table 3). The deviation of CPE-*ϕ*_1_ and CPE-*ϕ*_2_ parameter from day 1 can be related to physicochemical or geometrical inhomogeneities of the electrode surface [41].

### 3.4. Anodic Polarization Curves Study

**Figure 5:**
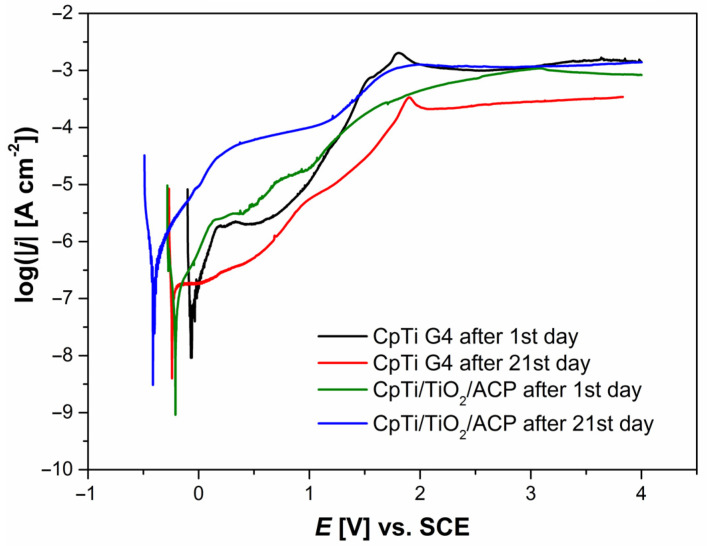
Anodic polarization curves at the polarization scan rate of *v* = 1 mV s^−1^ for the sandblasted and autoclaved CpTi G4 and CpTi G4|TiO_2_|ACP electrodes in the ASS at 37 °C after days 1 and 21 of immersion.

The effect of immersion time on the corrosion resistance of the sandblasted and autoclaved CpTi G4 and CpTi G4|TiO_2_|ACP electrodes in the ASS at 37 °C was determined on the basis of anodic polarization curves (Figure 5). Taking into account that the real potential differences in the human body do not exceed 2.5 V, the potential of 4 V was assumed as a sufficient anodic limit in the potentiodynamic measurements.

Analysis of the anodic polarization curves revealed their similar course. A minimum shift on the log|*j*| = f(*E*) curve toward cathodic potentials was visible for the CpTi G4|TiO_2_|ACP electrode in comparison with that for the CpTi substrate after days 1 and 21 immersion, which suggests lower corrosion resistance in the case of the substrate covered with ACP coating. The corrosion potential for CpTi G4 decreased during the immersion in the ASS from *E*_cor_ = −0.077(4) V after the test day 1 to *E*_cor_ = −0.119(2) V after the day 21 of testing. In the case of the CpTi G4|TiO_2_|ACP electrode, the *E*_cor_ changed more intensively from *E*_cor_ = −0.130(2) V on the first day of immersion to *E*_cor_ = −0.339(7) V on the last day of the test. For potentials with values lower than *E*_cor_, the tested electrodes showed resistance to corrosion. With potential values above *E*_cor_, the oxidation process began. On all of the anode polarization curves, a narrow passive range existed in the range of about 0.4(1)–1.2(2) V, in which the passive oxide layer on the surface of CpTi G4 electrode and the ECAD-derived ACP coating exhibited protective properties. The passive current densities of the order of 10^−6^ A cm^−2^ and 10^−5^ A cm^−2^ were observed for the CpTi G4 and CpTi G4|TiO_2_|ACP electrode after the 1st day of immersion, respectively. The observed passive current densities are typical for titanium and its alloys in the biological environment [23,24,39]. After day 21 of the test, the passive current density decreased in both cases by about one order of magnitude. After exceeding the passive range, an increase in the current densities with the increase of the anodic potential was observed until the maximum at which the transpassivation range was reached in the range of about 1.0(1)–1.9(2) V. The transpassive layers exhibited barrier properties up to 4 V, but the protection of the titanium substrate was provided with higher current densities of the order of 10^−3^ A cm^−2^ compared to the passive current density values. It should be noted that the recorded potentials are dependent on the polarization scan rate as anodic dissolution is a kinetically controlled process [42]. Pitting may be initiated by a slight surface defect, such as a scratch or local change in composition or damage to the protective coating. For potentials above the breakdown of the passive layers, an increase in the current density was observed with the increase in the anodic potential due to oxidation of metal cations forming the passive layers. 

**Figure 6:**
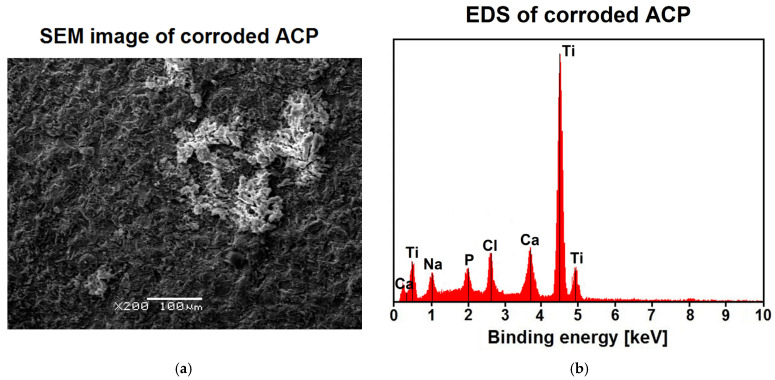
(**a**) SEM image of the ACP coating deposited at −3 V for 30 min at 20 °C after the potentiodynamic test performed after 21 days of immersion in the ASS at 37 °C; (**b**) Corresponding energy dispersive spectrum in the microregion of ACP coating.

Figure 6 shows the results of SEM/EDS study for the ACP coating deposited at −3 V for 30 min at 20 °C after the destructive potentiodynamic test performed after 21 days of immersion in the ASS at 37 °C. The SEM image reveals a slightly smoothed surface morphology as a result of anodic dissolution of the ACP coating, with an anodic potential limit of 4 V (Figure 5), as compared to the SEM image of the ACP coating before the corrosion test (Figure 1b). The ACP coating thickness decreased to 6.5(3) μm after the potentiodynamic test performed after 21 days of immersion in the ASS at 37 °C. As a result of the dissolution of the ACP coating and the transfer of the corrosion products in the form of ions into the neutral solution of the artificial saliva, the pH of the ASS changed to 6.68(12) after long-term corrosion resistance tests, which proves the increase in aggressiveness of the corrosive environment.

The results of EDS analysis in the microregion of the ACP coatings after corrosion test confirmed the presence of peaks originated from Ca and P elements forming the ACP coating and Ti coming from the substrate (Figure 6b). Other elements such as Na and Cl, which were present in the ASS and could be embedded into the surface of ACP coatings during long-term in vitro corrosion resistance studies, were also revealed. The change of composition ratio Ca:P in the ACP coatings was identified after corrosion test (Table 4). The average concentration of Ca and P decreased approximately five and seven times in comparison with the concentrations of these elements in the ACP coatings before corrosion studies, respectively (Table 1).

### 3.5. Work Function Study

**Figure 7:**
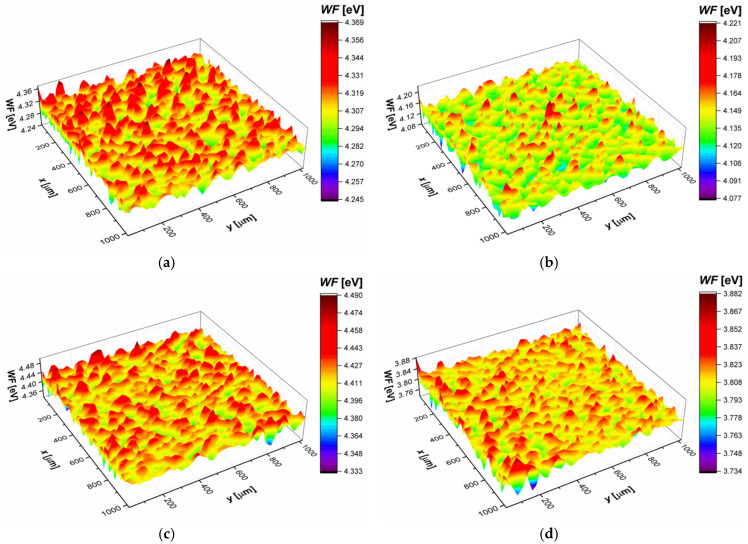
Work function (*WF*) maps: (**a**) Sandblasted and autoclaved CpTi G4 substrate before immersion in the ASS; (**b**) Sandblasted and autoclaved CpTi G4 after 21 days of immersion in the ASS; (**c**) ACP-coated CpTi G4 before immersion in the ASS; (**d**) ACP-coated CpTi G4 after 21 days of immersion in the ASS.

Work function maps of the sandblasted and autoclaved CpTi G4, as well as CpTi G4|TiO_2_|ACP before and after 21 days of immersion in ASS, are shown in Figure 7. Corresponding statistical parameters are gathered in Table 5.

It was found that the average work function (*WF*_av_) decreased by about 4% for the CpTi G4 substrate after 21 days of immersion in the ASS. In the case of ACP-coated CpTi G4, there was a decrease in *WF*_av_ by about 14%. A decrease in the *WF*_av_ value indicates a deterioration of the corrosion resistance of both investigated materials. However, greater susceptibility to corrosion was observed for the ACP-coated CpTi G4. It should be noted that the electric properties of the investigated materials correlate with their corrosion resistance (compare *WF*_av_, *E*_cor_, and *j*_cor_).

The effect of immersion in the ASS on the distribution of work function on the material surface was also investigated. The arithmetic mean deviation (*WF*_a_) and root mean square deviation (*WF*_rms_) determine the deviation of work function peaks/valleys heights in relation to the arithmetic average. Generally, both parameters, i.e., *WF*_a_ and *WF*_rms_, indicate decreasing heights of *WF* on the investigated surfaces after 21 days of immersion in the ASS. This result may be related to the partial dissolution of the surface peaks. Skewness (*WF*_sk_) and kurtosis (*WF*_ku_) provide information regarding the work function peaks/valleys profile as well.

For both types of investigated materials, the values of *WF*_sk_ indicate that, before immersion in the ASS, the work function peaks/valleys were symmetrically distributed around the average. However, after 21 days of immersion in the ASS on the sandblasted and autoclaved CpTi G4 surface, there was a predominance of peaks (*WF*_sk_ > 0), and on the ACP-coated CpTi G4 surface, there was a predominance of valleys (*WF*_sk_ < 0). Besides, the kurtosis values obtained for both materials indicate the presence of inordinately high peaks/deep valleys (*WF*_ku_ > 3). However, note that these discontinuities were much more pronounced in the case of ACP-coated CpTi G4. The obtained results show that the immersion in the ASS led to an increase in discontinuities of the CpTi G4 surface. However, their shape depended on the method of surface treatment.

## 4. Conclusions

Biomimetic CaP coatings were successfully deposited on the CpTi G4 substrate subjected to sandblasting and autoclaving using the ECAD method from the acetate bath at −3 V for 30 min at room temperature. The ECAD-derived CaP coatings were continuous, with a uniform distribution of chemical elements of Ca and P. EDS analysis of the CaP coatings revealed that the molar ratio of Ca:P was 1.5, which confirmed the presence of ACP. The obtained biomimetic ACP coatings were thin, with a thickness of 11.3(7) μm and porous with *R*a of 2.81(8) μm.

It was found that the corrosion potential of both ACP coatings and CpTi G4 substrate decreased during the immersion in the ASS at 37 °C for 21 days. Higher corrosion resistance was determined for the titanium substrate covered with a passive oxide layer. The ACP coatings and CpTi G4 substrate showed the passive-transpassive behavior and exhibited barrier properties up to 4 V.

The EIS study revealed the capacitive behavior of the ACP coatings and CpTi G4 with a high corrosion resistance. The mechanism and kinetics of pitting corrosion of the studied materials were determined based on the EIS measurements fitted by the equivalent electrical circuits of one- and two-CPE models. The decrease in the electron work function after 21 days of immersion in the ASS confirmed the electrochemical results.

## Figures and Tables

**Table 1:** The results of EDS analysis in the microregion of the amorphous calcium phosphate (ACP) coating deposited at −3 V for 30 min at 20 °C.

Element	Concentration (at.%)	Standard Deviation
Ca	45.932	(0.970)
P	30.920	(0.660)
Ti	23.148	(0.878)

**Table 2:** The parameters with standard deviations determined by approximation of the experimental EIS data for the sandblasted and autoclaved CpTi G4 electrode in the ASS at 37 °C and the equivalent electrical circuit model for the pitting corrosion process (see Figure 3e).

Immersion (Day)	*R*_1_ (Ω cm^2^)	CPE-*T*_1_ (F cm^−2^ s*^ϕ^*^−1^)	CPE-*ϕ*_1_	*R*_2_ (Ω cm^2^)
1	184(1)	3.03(33) × 10^−5^	0.834(30)	2.52(47) × 10^6^
2	184(1)	3.53(63) × 10^−5^	0.830(24)	8.42(71) × 10^5^
7	185(1)	3.44(74) × 10^−5^	0.780(26)	4.18(11) × 10^5^
14	187(2)	3.51(40) × 10^−5^	0.779(25)	1.42(29) × 10^5^
21	185(1)	4.19(13) × 10^−5^	0.740(45)	9.30(90) × 10^4^

**Table 3:** The parameters with standard deviations determined by approximation of the experimental EIS data for the CpTi G4|TiO_2_|ACP electrode in the ASS at 37 °C and the equivalent electrical circuit model for pitting corrosion process (see Figure 3e,f).

Immersion (Day)	*R*_1_ (Ω cm^2^)	CPE-*T*_1_ (F cm^−2^ s*^ϕ^*^−1^)	CPE-*ϕ*_1_	*R*_2_ (Ω cm^2^)	CPE-*T*_2_ (F cm^−2^ s*^ϕ^*^−1^)	CPE-*ϕ*_2_	*R*_3_ (Ω cm^2^)
1	36(2)	1.04(56) × 10^−5^	0.865(1)	2.55(21) × 10^5^	–	–	–
2	33(1)	1.65(13) × 10^−5^	0.832(2)	2.19(25) × 10^5^	–	–	–
7	33(1)	1.44(57) × 10^−5^	0.831(1)	1.64(29) × 10^5^	–	–	–
14	28(3)	1.99(47) × 10^−4^	0.860(4)	1.55(17) × 10^5^	8.30(18) × 10^−4^	0.720(9)	229(10)
21	29(4)	4.40(64) × 10^−4^	0.820(58)	2.58(15) × 10^4^	1.16(14) × 10^−3^	0.700(13)	152(12)

**Table 4:** The results of EDS analysis in the microregion of the ACP coating deposited at −3 V for 30 min at 20 °C after the potentiodynamic test performed after 21 days of immersion in the ASS at 37 °C.

Element	Concentration (at.%)	Standard Deviation
Ca	10.041	(0.650)
P	4.495	(0.451)
Ti	62.264	(0.835)
Na	14.022	(0.662)
Cl	9.178	(0.678)

**Table 5:** Statistical parameters calculated using work function (*WF*) maps of the sandblasted and autoclaved CpTi G4 and ACP-coated CpTi G4. *WF*_av_ is the arithmetic average, *WF*_a_ is the arithmetic mean deviation, *WF*_rms_ is the root mean square deviation, *WF*_sk_ is the skewness, and *WF*_ku_ is the kurtosis.

CpTi G4	*WF*_av_ (eV)	*WF*_a_ (eV)	*WF*_rms_ (eV)	*WF*_sk_	*WF*_ku_
sandblasted and autoclaved	4.31	14.2 × 10^−3^	18.0 × 10^−3^	−0.04	3.2
sandblasted and autoclaved after 21 days of immersion in artificial saliva	4.14	13.7 × 10^−3^	17.1 × 10^−3^	0.1	3.3
covered by ACP coating	4.42	14.5 × 10^−3^	18.6 × 10^−3^	−0.01	3.7
covered by ACP coating after 21 days of immersion in artificial saliva	3.81	11.8 × 10^−3^	15.2 × 10^−3^	−0.1	4.1

## Data Availability

The data presented in this study are available on request from the corresponding authors.
